# Outcomes of Emergent Isolated Coronary Bypass Grafting in Heart Failure Patients

**DOI:** 10.3390/life12122124

**Published:** 2022-12-16

**Authors:** Giacomo Bianchi, Edoardo Zancanaro, Rafik Margaryan, Giovanni Concistré, Egidio Varone, Simone Simeoni, Marco Solinas

**Affiliations:** 1 Department of Adult Cardiac Surgery, Ospedale del Cuore, Fondazione Toscana G. Monasterio, Via Aurelia Sud, 54100 Massa, Italy; 2 Department of Cardiac Surgery, Università Vita-Salute San Raffaele, Via Olgettina, 58, 20132 Milan, Italy

**Keywords:** emergent CABG, heart failure, outcomes

## Abstract

Patients with previously diagnosed HF are at greater risk for subsequent morbidity and mortality when hospitalized for an Acute Myocardial Infarction (AMI). The purpose of our study was to describe the time trend of the incidence of emergent CABG in patients with and without HF, the clinical characteristics, outcomes, and the risk factors for mortality of surgical revascularization in the short and medium term. This was a single-center retrospective observational study of patients who underwent isolated emergency CABG from January 2009 to January 2020. A propensity-score matching analysis yielded two comparable groups (n = 430) of patients without (n = 215) and with (n = 215) heart failure. In-hospital mortality did not differ in the two groups (2.8%; *p* > 0.9); the patients with heart failure presented more frequently with cardiogenic shock, and there was an association with mortality and mechanical circulatory support (OR 16.7–95% CI 3.31–140; *p* = 0.002) and postoperative acute renal failure (OR 15.9–95% CI 0.66–203; *p* = 0.036). In the early- and mid-term, heart failure and NSTEMI were associated with mortality (HR 3.47–95% CI 1.15–10.5; *p* = 0.028), along with age (HR 1.28–95% CI 1.21–1.36; *p* < 0.001). Surgical revascularization offers an excellent solution for patients with acute coronary syndrome, leading to a good immediate prognosis even in those with chronic heart failure.

## 1. Introduction

Heart failure (HF) is a complex clinical syndrome with symptoms and signs that result from any structural or functional impairment of ventricular filling or ejection of blood. Its prevalence is rapidly growing and is becoming a serious public health issue, a true “epidemic” [1]. Patients with previously diagnosed HF are at greater risk for subsequent morbidity and mortality when hospitalized for an acute myocardial infarction (AMI) in comparison with those who do not have this clinical syndrome [2,3]. Outcomes in HF patients presenting with AMI are improved by a strategy of early revascularization [4], but emergency CABGs are associated with significantly higher adverse outcomes [5]. In recent years, the proportion of emergent CABG in STEMI compared to NSTEMI hospitalization has decreased, but these patients have experienced a rise in noncardiac acute organ injury and cardiogenic shock [6]. The purpose of our study was to describe the time trend of the incidence of emergent CABG in patients with and without HF, the clinical characteristics, outcomes, and the risk factors for the mortality of surgical revascularization in the short and medium term.

## 2. Patients and Methods

### 2.1. Study Population

This was a single-center retrospective observational study of patients who underwent isolated emergency CABG from January 2009 to January 2020. The data were extracted from the Institutional Database and Reimbursement Registry, regarding patients with AMI as the primary diagnosis (International Classification of Diseases 9.0 Clinical Modification [ICD-9CM] 410.x and ICD-10CM I22.x-22.x). Patients with concomitant valvular disease warranting surgery were excluded as well as patients below 18 years of age. The emergent status of the operation was defined according to the STS criteria: patients who had ongoing, refractory, unrelenting cardiac compromise, with or without hemodynamic instability, who were not responsive to any form of therapy except cardiac surgery [7]. The population was divided into two groups, patients without pre-existing heart failure diagnosis (No HF) and patients with pre-existing heart failure diagnosis (HF). The definition of HF followed the most recent ESC 2021 guidelines [8].

### 2.2. Clinical Outcomes

Clinical outcomes included major events, such as 30-day death, cerebrovascular events, new-onset acute renal failure, new-onset atrial fibrillation, bleeding requiring surgical re-exploration, and also the main postoperative features of surgical patients.

### 2.3. Data Collection and Followup

Data were acquired anonymously from the institutional database. Due to the retrospective nature of the study, the IRB waived the informed consent.

### 2.4. Statistical Analysis

Data are presented as the mean ± standard deviation for normal or the median for nonnormal distribution. Continuous variables were tested with the Kolmogorov–Smirnov test for normality. Categorical variables are given as frequencies and percentages. The Wilcoxon rank sum test, Pearson’s chi-square test, and Fisher’s exact test were used to compare the categorical variables between patients with heart failure and those without heart failure. To reduce the treatment selection bias and potential confounding factors and to adjust for significant differences in patient characteristics, the propensity score-matching was performed.

The propensity scores were estimated using a multivariate logistic regression model for receiving emergent CABG in patients with and without heart failure. All baseline characteristics were entered to calculate the propensity score. A local optimal algorithm with the caliper method was used for the development of the propensity score-matched pairs without replacement (1:1 match), (area under the curve of 0.78). A matching caliper of 0.2 standard deviations of the logit of the estimated propensity score was enforced to ensure that matches of poor fit were excluded. The matching procedure was performed by using the R MatchIt package (R Development Core Team, R Foundation for Statistical Computing, Vienna, Austria). After the propensity score-matching, the covariates were compared as described before.

Univariate logistic regression analysis was performed to assess the association between the patient baseline characteristics and postoperative features with in-hospital death. All variables with *p* < 0.2 entered the multivariable logistic regression analysis to identify an association with in-hospital death.

Survival analysis was conducted using the Kaplan–Meier method, and comparison by the presence of heart failure (No HF vs. HF) was tested using the log-rank test.

Statistical significance was assumed when the null hypothesis could be rejected at *p* < 0.05. All P values reflect the results of two-sided tests. Statistical analyses were conducted using R (version 3.6.2).

## 3. Results

A total of 891 patients underwent CABG in the study period. We focused our attention on patients that already had a diagnosis of heart failure (n = 215), and we compared them to patients without heart failure (n = 676) (Table 1).

### 3.1. Propensity Score-Matched Population Analysis

Because of the significant imbalance of covariates in the two groups, the use of propensity score-matching produced two homogeneous and comparable groups, each consisting of 215 patients (Table 2).

#### 3.1.1. Baseline Characteristics

**Figure 1:**
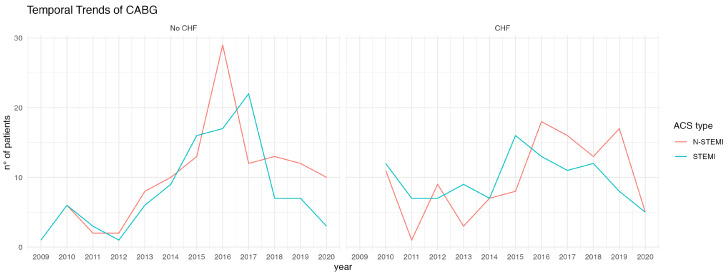
Temporal trend of patients with ACS treated by surgical revascularization (CABG). The left side of the figure outlines the increasing trend of patients with ACS without CHF with a prevalence of NSTEMIs; this trend is also present in the group of patients with CHF, where NSTEMIs have been more frequent over the years. Also noted is the decrease of cases in the year 2020, due to the SARS-CoV2 pandemic.

The time trend of the patients who were referred to our center for surgical revascularization due to ACS is shown in Figure 1. The patients had a mean age of 71 years and were predominantly male. Most patients (82% in both groups) had a moderately reduced or reduced ejection fraction. There was an equal distribution of ACS type in the two groups (*p* = 0.4). Approximately one-third of the patients required CABG surgery within the first 6–24 h after diagnosis (No CHF vs. HF 33% vs. 33%), while in most cases surgery was performed within a time window of 1–7 days. In less than 10% of patients, surgery was performed within 6 h (No HF vs. HF 4.7% vs. 8.4%; *p* = 0.5). In most cases (53% in both groups), there was coronary three-vessel disease.

#### 3.1.2. Operative Outcomes

The majority of patients had the procedure performed with the use of cardiopulmonary bypass (CPB) (No HF vs. HF, 96.7% vs. 97.2%, *p* = 0.8); given the emergent situation and the average age of the patients, although there was multivessel coronary artery disease in more than 70% of patients, only one internal thoracic artery was used (No HF vs. HF, 82% vs. 86%, *p* = 0.3). Revascularization in the two groups (No HE vs. HF) was complete in 60% and 54% of cases, respectively, with no statistically significant differences (Table 3).

#### 3.1.3. Postoperative Outcomes

Patients undergoing emergency surgical revascularization with previous diagnosis of HF required mechanical support for the circulation (IABP, VA-ECMO or the combination of both) more frequently compared with the other group (No HF vs. HF, 14% vs. 25%, *p* = 0.005).

Patients with HF presented in the postoperative period a higher incidence of new-onset atrial fibrillation (0.9% vs. 4.2%, *p* = 0.033), as well as a longer intensive care and hospital stay; no differences were observed in hospital mortality, which stood at 2.8% in both groups (*p* > 0.9) (Table 4).

#### 3.1.4. Risk Factors for In-Hospital Mortality

Factors associated with in-hospital mortality were found to be (Table 5) presentation with cardiogenic shock (*p* < 0.001), need for mechanical circulatory support (*p* < 0.001), and STEMI-type ACS, the latter trending toward statistical significance (*p* = 0.069); among intraoperative factors, time to CPB (*p* = 0.002) and time to aortic cross-clamp (*p* = 0.006) were associated with mortality. At multivariable analysis (Table 6), the need for mechanical circulatory support (OR 16.7–95% CI 3.31 to 140; *p* = 0.002) and the onset of postoperative acute renal failure (OR 15.9–95% CI 0.66-203; *p* = 0.036) were significantly associated with in-hospital mortality.

#### 3.1.5. Early- and Mid-Term Outcomes

**Figure 2:**
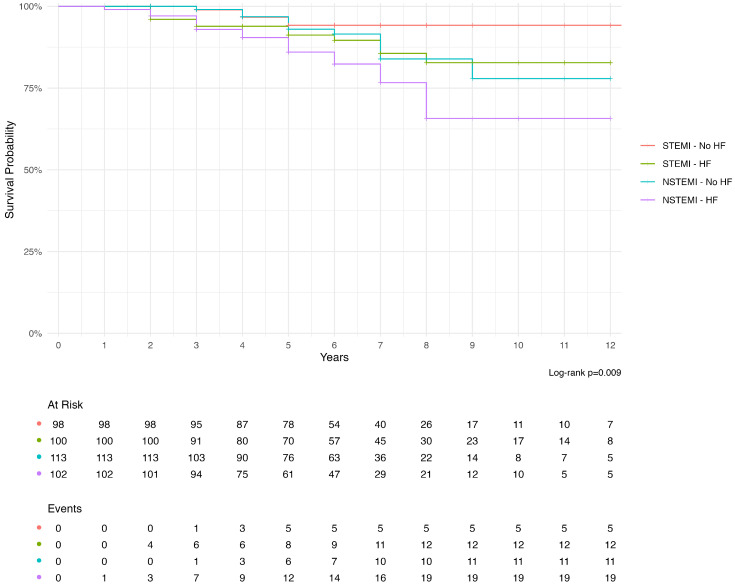
Survival of patients operated on with emergent CABG according to the preoperative diagnosis of chronic heart failure. The patients with heart failure had significantly lower survival compared to the control group; furthermore, the worst prognosis was for patients with chronic heart failure and the NSTEMI type of acute coronary syndrome.

The median followup was 6 years (min 1, max 12 years). Mortality was higher in the group diagnosed with chronic heart failure (0.5%, 6.6%, 11%, and 25%) than in the control group (0%, 1%, 6.4%, and 14%) at 1, 3, 5, and 12 years, respectively (*p* = 0.013). Figure 2 shows the survival according to the type of ACS and the presence of HF; of note, the patients with chronic heart failure and NSTEMI had the worst mid-term prognosis.

The probability of death at followup was higher in patients who presented with an N-STEMI type of ACS from year 5 (*p* = 0.031), as shown in Table 7; furthermore N-STEMI in the context of chronic heart failure was a risk factor for mid-term mortality (HR 3.47–95% CI 1.15–10.5; *p* = 0.028) along with age (HR 1.28–95% CI 1.21–1.36; *p* < 0.001).

## 4. Discussion

Ischemic coronary artery disease (CAD) remains the leading cause of mortality globally due to sudden death and heart failure (HF). In recent years, the number of patients undergoing coronary angiography increased substantially per year driven as indicated by HF, more than angina pectoris or STEMI [9].

In a recent National Inpatient Sample (NIS) analysis of the time trend and outcome of patients undergoing emergency surgical revascularization, the incidence of chronic heart failure on admission did not change in the three eras examined, ranging from 28.1% to a 30.7% [6].

The cornerstone of therapy in acute coronary syndromes is early revascularization [4,5,10].

Revascularization with CABG is associated with better long term prognosis, lower incidence of cardiovascular death and myocardial infarction, along with less repeated revascularization, compared to percutaneous interventions (PCI) [11]. The superiority of CABG over PCI is particularly evident in patients with severely reduced left ventricular function [12].

In this retrospective work we compared, using propensity-score matching, a sample of patients diagnosed with chronic heart failure compared with a control sample who needed emergent surgical revascularization.

We saw that the effect was a good immediate outcome with low mortality (2.8%) in both groups, which were comparable to each other.

This finding was certainly an improvement over data from studies reporting an in-hospital mortality of between 7.4% and 8.7% [4,10] and others reporting a progressive decline in such mortality in more recent years but with an overall figure of 6.6% [5].

In our study, patients with chronic heart failure had almost twice the incidence of cardiogenic shock; this figure correlated with the observed increased need for mechanical circulatory support (MCS), up to 25%.

This finding correlated with those in the large study by Patlolla et al., where the incidence of cardiogenic shock in patients undergoing emergency CABG has increased over time, from 6.4% to 11.5%, with mechanical circulatory support being used in about 20% of subjects [6]. Furthermore, therapies to provide hemodynamic support are increasingly being used with success in the context of acute myocardial infarction [13].

This occurrence identified these patients as an increased high-risk group, as evidenced by the significant association of MCS with in-hospital mortality. The very nature of the acute coronary syndrome, associated in some patients in our study with cardiogenic shock with the need for peri- and postoperative circulatory support, resulted in a higher critical status after surgery; in fact, these patients had a higher incidence of acute renal failure, both from low flow and related to MCS, with longer ICU and hospital stay times. These data were in keeping with other studies in which cardiogenic shock was burdened with high mortality and correlated with the onset of renal failure and atrial fibrillation [14].

An interesting finding from the analysis at followup is that patients with chronic heart failure had a higher mortality, already evident from the third year onward; this trend continued steadily upward, until it almost doubled at 12 years.

When we considered the type of acute coronary syndrome, we saw a bimodal mortality trend. In the immediate perioperative period, presentation with STEMI had a trend of association with mortality; the achieved nonstatistical significance was probably due to the presence in our center’s territory of a cardiology network for heart attack that leads to early diagnosis and equally rapid revascularization [15].

This is in contrast to other work that has identified STEMI as a factor associated with in-hospital mortality [16].

Following an established trend in recent years, NSTEMI are increasing and, given the characteristics of the greater chronic morbidity of these patients, their prognosis is also worse [3,4]. In fact, we observed a stable increase in the mortality of such subjects as early as the fifth year post-event to more than double at the end of the followup period considered. This trend is driven by the unfavorable prognosis in the medium term of patients with chronic heart failure and acute coronary syndrome NSTEMI, whose association has a greater negative weight than age itself in the determinism of the death event.

## 5. Limitations and Future Directions

The present study had as limitations the retrospective observational nature in which the imbalance of covariates was mitigated by the use of propensity-score matching; this method, although commonly agreed upon and effective, significantly reduced the sample size and inherently resulted in data loss in order to achieve “pseudo-randomization”.

The choice to refer patients for emergency revascularization depends heavily on the Heart Team’s discussion performed and the professionals involved. In future studies, it will be necessary to determine on the basis of these data as well which timing is most appropriate for surgical revascularization in the context of AMI.

The study also considered the period of onset of the Sars-CoV2 pandemic, which drastically reduced the sample size and thus, although this trend was common worldwide, it may have partially affected the results on the time course. With regard to followup, a mortality analysis was conducted based on the census status of the patients considered; other events such as heart failure recurrence as a risk factor for mortality were not considered; this event was beyond the scope of the present study but of high interest, and it will need to be addressed in future studies, also based on the results of the present scientific work.

## 6. Conclusions

Surgical revascularization offers an excellent solution for patients with acute coronary syndrome, leading to a good immediate prognosis even in those with chronic heart failure. Patients with NSTEMI and chronic heart failure turn out to be a fragile population and particularly at risk in the short to medium term.

## Figures and Tables

**Table 1:** Baseline characteristics of the unmatched populations.

Characteristic	No HF, N = 676 ^1^	HF, N = 215 ^1^	*p*-Value ^2^
Age	69 (61, 77)	71 (62, 78)	0.074
Male	537 (79%)	174 (81%)	0.6
Hypertension	495 (73%)	148 (69%)	0.2
Diabetes mellitus type II	152 (22%)	53 (25%)	0.5
Dyslipidemia	309 (46%)	63 (29%)	<0.001
Atrial fibrillation	16 (2.4%)	3 (1.4%)	0.6
Active smoker	93 (14%)	21 (9.8%)	0.13
COPD	80 (12%)	18 (8.4%)	0.2
Kidney disease			0.017
>85 mL/min	212 (31%)	46 (21%)	
50–85 mL/min	367 (54%)	137 (64%)	
<50 mL/min	97 (14%)	32 (15%)	
Cardiogenic shock	9 (1.3%)	17 (7.9%)	<0.001
Previous valve surgery	2 (0.3%)	0 (0%)	>0.9
Previous CABG	1 (0.1%)	0 (0%)	>0.9
Previous PCI	49 (7.2%)	6 (2.8%)	0.018
Previous AMI	37 (5.5%)	6 (2.8%)	0.11
LV function			<0.001
Normal; EF >= 50%	400 (59%)	37 (17%)	
Moderately reduced, EF 41–49%	198 (29%)	90 (42%)	
Reduced, EF <= 40%	78 (12%)	88 (41%)	
ACS type			<0.001
N-STEMI	449 (66%)	108 (50%)	
STEMI	227 (34%)	107 (50%)	
Time from MI to surgery			0.003
<6 h	28 (4.1%)	18 (8.4%)	
6-24 h	160 (24%)	70 (33%)	
1–7 days	459 (68%)	117 (54%)	
8–21 days	27 (4.0%)	9 (4.2%)	
>21 days	2 (0.3%)	1 (0.5%)	
Number of diseased vessels			0.4
1	117 (17%)	30 (14%)	
2	172 (25%)	51 (24%)	
3	342 (51%)	115 (53%)	
>= 4	45 (6.7%)	19 (8.8%)	

^1^ Median (IQR); n (%). ^2^ Wilcoxon rank sum test; Pearson’s chi-square test; Fisher’s exact test.

**Table 2:** Baseline characteristics of propensity score-matched population.

Characteristic	No HF, N = 215 ^1^	HF, N = 215 ^1^	*p*-Value ^2^
Age	71 (62, 79)	71 (62, 78)	>0.9
Male	171 (80%)	174 (81%)	0.7
Hypertension	147 (68%)	148 (69%)	>0.9
Diabetes mellitus type II	57 (27%)	53 (25%)	0.7
Dyslipidemia	70 (33%)	63 (29%)	0.5
Atrial fibrillation	5 (2.3%)	3 (1.4%)	0.7
Active smoker	25 (12%)	21 (9.8%)	0.5
COPD	21 (9.8%)	18 (8.4%)	0.6
Kidney disease			>0.9
>85 mL/min	44 (20%)	46 (21%)	
50–85 mL/min	136 (63%)	137 (64%)	
<50 mL/min	35 (16%)	32 (15%)	
Cardiogenic shock	9 (4.2%)	17 (7.9%)	0.11
Previous valve surgery	0 (0%)	0 (0%)	
Previous CABG	0 (0%)	0 (0%)	
Previous PCI	7 (3.3%)	6 (2.8%)	0.8
Previous AMI	9 (4.2%)	6 (2.8%)	0.4
LV function			0.15
Normal; EF >= 50%	39 (18%)	37 (17%)	
Moderately reduced, EF 41–49%	107 (50%)	90 (42%)	
Reduced, EF <= 40%	69 (32%)	88 (41%)	
ACS type			0.4
N-STEMI	117 (54%)	108 (50%)	
STEMI	98 (46%)	107 (50%)	
Time from MI to surgery			0.5
<6 h	10 (4.7%)	18 (8.4%)	
6–24 h	70 (33%)	70 (33%)	
1–7 days	121 (56%)	117 (54%)	
8–21 days	13 (6.0%)	9 (4.2%)	
>21 days	1 (0.5%)	1 (0.5%)	
Number of diseased vessels			0.9
1	33 (15%)	30 (14%)	
2	45 (21%)	51 (24%)	
3	115 (53%)	115 (53%)	
>= 4	22 (10%)	19 (8.8%)	

^1^ Median (IQR); n (%). ^2^ Wilcoxon rank sum test; Pearson’s chi-square test; Fisher’s exact test.

**Table 3:** Operative characteristics of the propensity score-matched population.

Characteristic	No HF, N = 215 ^1^	HF, N = 215 ^1^	*p*-Value ^2^
CPB time (min)	102 (84, 124)	106 (90, 126)	0.084
Cross-Clamp time (min)	65 (53, 79)	65 (54, 80)	0.8
OPCAB	8 (3.7%)	6 (2.8%)	0.6
Single ITA used	176 (82%)	184 (86%)	0.3
Double ITA used	28 (13%)	20 (9.3%)	0.2
Complete revascularization	129 (60%)	116 (54%)	0.2
Mechanical circulatory support	31 (14%)	54 (25%)	0.005

^1^ Median (IQR); n (%). ^2^ Wilcoxon rank sum test; Pearson’s chi-square test 3 OPCAB: Off-pump coronary artery bypass; ITA: internal thoracic artery.

**Table 4:** Post-operative outcomes.

Characteristic	No HF, N = 215 ^1^	HF, N = 215 ^1^	*p*-Value ^2^
TIA/Stroke	0 (0%)	0 (0%)	
New-onset renal failure	7 (3.3%)	2 (0.9%)	0.2
Bleeding requiring rethoracotomy	0 (0%)	0 (0%)	
New onset atrial fibrillation	2 (0.9%)	9 (4.2%)	0.033
ICU stay (days)	1.00 (1.00, 1.00)	1.00 (2.00, 3.00)	<0.001
Hospital stay (days)	9.0 (7.0, 12.0)	10.0 (7.0, 14.0)	0.023
In-hospital death	6 (2.8%)	6 (2.8%)	>0.9

^1^ n (%); Median (IQR). ^2^ Fisher’s exact test; Pearson’s chi-square test; Wilcoxon rank sum test.

**Table 5:** Univarite logistic regression for in-hospital mortality.

Characteristic	N	OR ^1^	95% CI ^1^	*p*-Value
STEMI	430	3.40	1.00, 15.5	0.069
Cardiogenic shock	430	9.00	2.26, 31.0	<0.001
Male	430	0.48	0.15, 1.84	0.2
Moderately reduced, EF 41–49%	430	2.42	0.75, 9.19	0.2
CABG timing 6–24 h	430	2.12	0.65, 6.89	0.2
CPB time (min)	430	1.02	1.01, 1.03	0.002
Cross-clamp time (min)	430	1.02	1.01, 1.04	0.006
Complete revascularization	430	2.31	0.68, 10.5	0.2
Mechanical circulatory support	430	22.9	5.88, 151	<0.001
Postoperative ARF	430	4.66	0.24, 28.8	0.2

^1^ OR = Odds Ratio, CI = Confidence Interval.

**Table 6:** Multivariable logistic regression for in-hospital mortality.

Characteristic	OR ^1^	95% CI ^1^	*p*-Value
STEMI	2.58	0.60, 15.0	0.2
Cardiogenic Shock	3.02	0.67, 12.4	0.13
Post-Operative ARF	15.9	0.66, 203	0.036
CPB time (min)	1.01	1.00, 1.04	0.3
Cross-clamp time (min)	1.02	0.98, 1.05	0.4
Mechanical circulatory support	16.7	3.31, 140	0.002

^1^ OR = Odds ratio, CI = Confidence interval.

**Table 7:** Cumulative incidence function of death according to ACS type.

Characteristic	1-Year	2-Year	5-Year	10-Year	12-Year	*p*-Value ^1^
ACS ^2^						0.031
N-STEMI	0.47%	1.4%	10%	28%	28%	
	(0.04%, 2.4%)	(0.38%, 3.8%)	(6.3%, 15%)	(18%, 39%)	(18%, 39%)	
STEMI	0.00%	2.0%	7.3%	12%	12%	
	(—%, —%)	(0.67%, 4.8%)	(4.1%, 12%)	(6.9%, 19%)	(6.9%, 19%)	

^1^ Gray’s test. ^2^ Acute coronary syndrome.

## Data Availability

Not applicable.

## Abbreviations

The following abbreviations are used in this manuscript:

ACS	Acute Coronary Syndrome
HF	Heart Failure
CHF	Congestive Heart Failure
AMI	Acute Myocardial Infarction
STEMI	ST-Elevation Myocardial Infarction
NSTEMI	Non ST-Elevation Myocardial Infarction
TIA	Transitory Ischemic Attack
ARF	Acute Renal Failure
ITA	Internal Thoracic Artery
OPCAB	Off-Pump Coronary Artery Bypass
ICU	Intensive Care Unit
ECMO	Extra-Corporeal Membrane Oxygenation
IABP	Intra-Aortic Balloon Pump
CPB	Cardio-Pulmonary Bypass
PCI	Percutanteous Coronary Intervention
MCS	Mechanical Circulatory Support
